# Paediatric Restrictive Cardiomyopathy - Diagnosis and Challenges: A report of two cases

**DOI:** 10.18295/squmj.1.2024.001

**Published:** 2024-05-27

**Authors:** Dalal S Husain, Niranjan P Joshi, Khalfan S Al Senaidi, Hilal Al Riyami

**Affiliations:** 1 Sultan Qaboos University, Muscat, Oman; 2 Child Health Department, Sultan Qaboos University Hospital

**Keywords:** Cardiomyopathy, Restrictive Cardiomyopathy, Congestive Heart Failure

## Abstract

Restrictive cardiomyopathy is one of the rarest forms of cardiomyopathies in paediatric patients characterised by impaired myocardial relaxation or compliance with restricted ventricular filling, leading to a reduced diastolic volume with a preserved systolic function. We report 2 cases—a 5-year-old boy who presented with abdominal distension and palpitation with family history of similar complaints but no definite genetic diagnosis as yet and a 5-year-old girl who presented with chronic cough and shortness of breath. Both cases were diagnosed in a tertiary care hospital in Muscat, Oman, in 2019 and are managed supportively with regular outpatient follow-up. This is the first series of reported cases of paediatric restrictive cardiomyopathy from Oman.

Restrictive cardiomyopathy (RCM) is one of the rarest forms of cardiomyopathies in paediatric patients, with an overall prevalence of 2–5% of all types of cardiomyopathies.^1^ Functionally, RCM is characterised by impaired myocardial relaxation or compliance with restrictive filling, leading to a reduced diastolic volume with a preserved systolic function.^2^ This leads to atrial dilatation, which is represented by the large P-waves on an electrocardiogram (ECG) and bundle branch block.^1^ Findings in an ECG illustrate atrial dilatation and small ventricles, with an element of atrioventricular regurgitation that worsens the atrial enlargement.^1^ The causes of RCM are divided into primary and secondary, which are subdivided into familial/sporadic causes and systemic disorders, respectively.^3^ Secondary causes are usually seen in adults and include systemic infiltrative diseases like amyloidosis, Gaucher’s disease and storage disorders such as Fabry’s disease and others including scleroderma, endomyocardial fibrosis and carcinoid syndrome.^3^ RCM can be confused with constrictive pericarditis and is challenging to differentiate clinically or with imaging; however, it is important to differentiate them as constrictive pericarditis can be treated surgically, whereas RCM has a high mortality rate.^1^ The reported complications of RCM include heart failure and arrhythmias compared to the rarer complications including thromboembolism and increased pulmonary vascular resistance.^4^

We report 2 patients who presented at a tertiary care hospital in Muscat, Oman, with RCM at an early age of 5 years—one male with abdominal distension and palpitations and one female with chronic cough and shortness of breath.

## Case Reports

### CASE 1

A 5-year-old previously healthy boy presented with history of gradually worsening abdominal distension of 10-days duration, which was associated with palpitations. He had a history of recurrent fever and upper respiratory tract infections requiring intravenous antibiotics in a private clinic for 2 days. The mother also gave history of decreased appetite with poor weight gain over the past 4 months. The rest of his history was unremarkable.

He is the fourth child born to a first-degree consanguineous parents with 5 other siblings; 2 of his brothers had expired with a similar presentation. His oldest brother passed away at the age of 24 years after being admitted with abdominal distension, the details of which are not available. He was suspected to have RCM. The older brother had other medical problems including atrial flutter, hypothyroidism, liver cirrhosis and hypogonadism, for which he had also received testosterone briefly. The second brother, who had also expired, had developed abdominal distension at the age of 5 years and passed away at 12 years of age with no definite diagnosis. No other family history of cardiac diseases or sudden deaths were noted in the family.

On assessment, this child looked active, pale, with no dysmorphic features. His weight was 15 kg (<10^th^ percentile) and height was 106 cm (25^th^ percentile). He was stable on admission and cardiac examination revealed a normal S1 and loud S2. The chest was clear with bilateral normal breath sounds. The abdomen was distended on inspection with visible abdominal veins. There was a non-tender hepatomegaly of 7 cm below the right costal margin and no splenomegaly. The rest of the examination was normal. As a result, he was first checked by the gastroenterology team and was referred to cardiology once gastro-intestinal causes were ruled out.

Laboratory findings are illustrated in Table 1. Abdominal ultrasound revealed enlarged echogenic coarse liver suggestive of liver parenchymal disease and congestive hepatomegaly. Hepatic veins and intrahepatic inferior *vena cava* were dilated. A chest X-ray was done on admission and was unremarkable.

**Figure 1:**
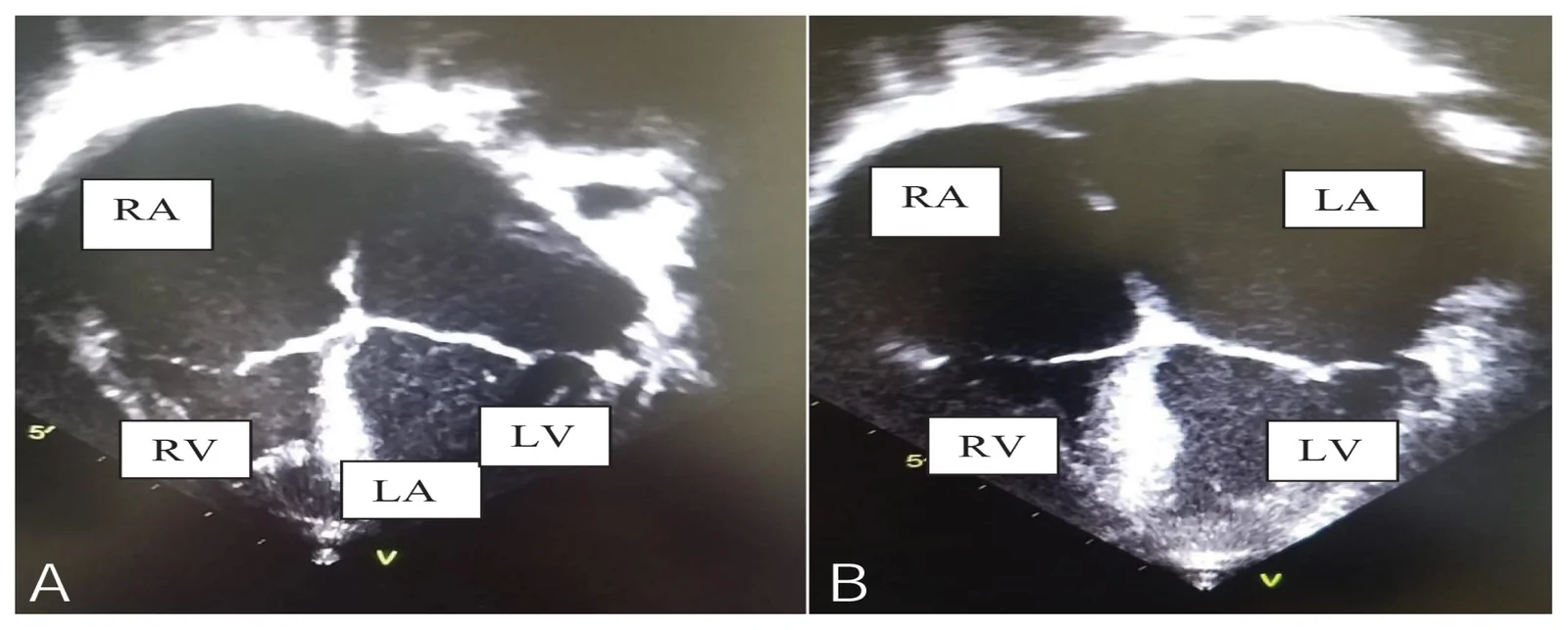
Echocardiography of **(A)** case 1 and **(B)** case 2 that presented to a tertiary care hospital in Muscat, Oman in 2019. RA = right atrium; LA = left atrium; RV = right ventricle; LV = left ventricle.

ECG findings are shown in Table 2. The echocardiography showed severely dilated right and left atrium with severe tricuspid valve regurgitation along with a trivial mitral regurgitation. It also illustrated mildly reduced systolic function with ejection fraction of 50% and the left ventricle showed apical trabeculations with features suggestive of non-compaction. There was no pericardial effusion. The detailed findings of the echocardiography are shown in Table 3 and Figure 1. The 24-hour Holter ECG was normal.

He was started on furosemide, spironolactone and digoxin. Currently, he is followed-up with cardiology every 6–8 months and has had 2 admissions since diagnosis for chest infections. Consent for publication was obtained from the patient’s guardian.

### CASE 2

A 5-year-old previously healthy girl presented with a 4-day history of cough and poor oral intake. There was no history of fever, no shortness of breath and no exposure to sick contacts. She had a history of night sweats and palpitations that were aggravated by change of posture. There was no history of chest pain, cyanosis or syncope. She had a similar episode 1 month prior and was treated symptomatically elsewhere. Her father reported a history of easy fatigability with running as compared to his other children and also poor appetite and poor weight gain for the past 2 years. There is history of inability to sleep lying flat and needing head elevation for the last few months. She had cataract surgery at the age of 3 years.

She is the eldest child of a first-degree consanguineous parents. One of her paternal cousins had a cardiac defect that needed catheterisation, but no details were available. She also had a maternal aunt who developed valvular heart disease at the age of 12 years and required valve replacement. She has 3 other younger siblings who are doing well. There is no history of other cardiac disease in the family.

On physical assessment, the child was in respiratory distress with mild recessions and tachypnea up to 30 breaths per minute. Her weight was 14.75 kg (10^th^ percentile) and her height was 114.5 cm (10^th^ percentile). She had periorbital edema with hypertelorism and clubbing. A chest examination revealed bilateral basal scattered crepitations. Cardiac examination revealed normal heart sounds, with gallop and a pansystolic murmur grade II/VI best heard at the apex. Abdominal examination revealed distension and tender hepatomegaly of 8–9 cm below the right costal margin.

Laboratory findings are depicted in Table 1. A chest X-ray showed cardiomegaly with congested lungs and right para-cardiac haziness. ECG and echocardiography findings and diagram are shown in Tables 2 & 3 and Figure 1, respectively. Her initial working diagnosis was multisystem inflammatory syndrome in children (MISC) causing acute heart failure. She also had a full septic work-up—to rule out pneumonia, pleural effusion and myocarditis—and was initially started on furosemide and spironolactone. During her further admissions, digoxin, captopril and aspirin were added gradually. The genetic and metabolic teams were involved to exclude secondary causes. At the last follow-up, she was intermittently in atrial flutter-fibrillation needing higher doses of digoxin for rate control. Consent for publication was obtained from the patient’s guardian.

## Discussion

RCM is one of the common causes of adult diastolic heart failure, which could be explained by the different risk factors affecting this age group.^5^ In contrast, these risk factors are absent in the paediatric age group, making this type of cardiomyopathy a rare occurrence in children with incidence of 0.04 per 100,000 in the USA.^6^ It is mostly diagnosed between the ages of 6–10 years, corresponding with the current cases, where both patients were 5 years of age.^5^ Both current cases were idiopathic.

As per the American Heart Association (AHA), the most common mode of inheritance is autosomal dominant.^7^ In case 1, the 5-year-old boy also had a strong family history of cardiac diseases and sudden deaths suggesting autosomal dominant inheritance, though no genetic diagnosis was confirmed until the write-up of this report.

RCM presents with a wide variety of symptoms, making the diagnosis even more difficult. In different case studies, a 10-year-old boy collapsed as he was playing football and was found to have a large liver and high B-type natriuretic peptide, along with abnormal echocardiography findings, consistent with RCM.^5^ Both the current patients presented with hepatomegaly, along with cough and fever. Similar to the current cases, the patient in Denfield’s study also had recurrent respiratory illnesses.^5^ These presentations are mostly due to the high filling pressures that cause pulmonary edema, pulmonary hypertension, hepatomegaly and peripheral edema.^5^ On the other hand, a case reported from Saudi Arabia reported an 11-year-old girl who presented with lower limb swelling and paraesthesia with no chest pain or shortness of breath, diagnosed with thromboembolism and RCM and treated with cardiac transplant.^8^ Therefore, the non-specific signs and symptoms of RCM may lead to an initial diagnosis of different respiratory or alimentary illnesses and the cardiac diagnosis may be missed or delayed as in the current 2 cases.

The first case was admitted under general paediatrics and the initial assessment was performed by the gastroenterology team. Once the gastroenterology causes were ruled out, the patient was referred to the cardiology team. The main cause of delay in the diagnosis was the presentation with abdominal distension with hepatomegaly, hence causes such as liver diseases and malignancies were ruled out before looking for other causes.

The second case had chronic non-specific cough for the last 2–3 months. She was seen elsewhere by general paediatrician and was treated for acute chest infection versus asthma. The chest X-ray done outside showed borderline cardiomegaly which was missed as were the important details in history, such as worsening inability to lie flat and easy fatigability.

Both cases presented with non-cardiac symptoms which led to a delay in the diagnosis. These cases underscore the importance of a good history and physical examination and the need to approach patients with chronic complaints with a wider frame of mind.

The diagnosis of RCM can be done by utilising the ECG, echocardiography and cardiac MRI, if needed. The main finding as per the AHA, is the biatrial enlargement on echocardiography and surface ECG with preserved systolic function.^7^

Commenting on the diastolic function in the paediatric age group may be difficult due to the variability of presentation or the need for sedation.^8^ Echocardiography can also differentiate between RCM and constrictive pericarditis (CP), which changes the management completely.^9^ In CP, the chamber compliance is reduced due to external pressure, causing an increased interventricular dependence and irregularity between intracardiac and intrathoracic pressure during respiration as shown by doppler echocardiography along with septal shifting.^10^ More specifically, annular tissue doppler can further distinguish the two entities. In RCM, the early diastolic velocity of mitral annulus is reduced, whereas it is normal or increased in CP.^10^ Cardiac catheterisation shows similar features in both diseases including early rapid diastolic filling with elevated end-diastolic pressures. The main finding to differentiate CP from RCM is the respiratory variation in pressures.^10^ Biopsy of the endocardium in children with RCM is not specific and is not helpful in making the diagnosis.^9^

The prognosis of RCM is generally poor, with a survival rate of approximately 2 years from the day of diagnosis.^11^ Management of RCM is mainly symptomatic, involving diuretics in pulmonary congestion, pacemakers in arrhythmia, and anticoagulants in a thromboembolic event.^12^ The use of diuretics should be carefully assessed as they are preload dependent and should not be dried.^12^ There is no proven role for digoxin and beta-blockers; however, it might be helpful with tachyarrhythmia or heart rate control.^5^^,^^13^ The definite therapy is a cardiac transplant which showed the 10-year survival rate post-transplant to be similar to other types of cardiomyopathies.^12^ The outcome of transplant has improved with a median graft half-life of 12 years.^11^ The question of when to send such cases for heart transplantation is still controversial. However, since medical therapy is just symptomatic many centres post these cases for transplant immediately after diagnosis.^11^ The final decision lies with the institute itself and their own criteria.^13^ A case series from Spain reported 9 cases of RCM, of which 5 underwent cardiac transplant with at least 4-years survival post-transplant.^3^ The need for heart transplant was not discussed in the current cases due to the non-availability of this management option in Oman.

## Conclusion

RCM in children is a rare entity with no cases reported in Oman till date. Proper symptomatic management is essential in children with RCM and, most importantly, a timely heart transplant to prevent sudden cardiac death as well as irreversible pulmonary hypertension. In Oman, there is a need for a national programme for heart transplant to help children with such diseases.

## Figures and Tables

**Table 1:** Laboratory findings of two cases that presented to a tertiary care hospital in Muscat, Oman in 2019.

Findings	Case 1	Case 2	Normal range
C-reactive protein in mg/L	13	23	0–5
Creatine kinase in U/L	Normal	Normal	26–192
Ck-MB in U/L	32.9 Abnormal	43.2 Abnormal	0–25
Troponin-T in ng/L	Normal	68 Abnormal	<14
Pro-BNP in pg/ mL	2,079 Abnormal	Not done (No reagent)	20–160
TFT (T4 in pmol/L; TSH in mIU/L)	Abnormal TSH (sick euthy)	Normal	T4: 11.5–28.3; TSH: 0.72–11
ANA	Negative	Negative	-
Endomyocardial panel	WES negative	CRYAB mutation	-
COVID-19 swab	Negative	Negative	-

CK-MB = creatine kinase-myoglobin binding; Pro-BNP = Pro-B-type natriuretic peptide; TFT = thyroid function test; ANA = Antinuclear antibody test; WES= whole exome sequencing; CRYAB= Crystallin alpha B; COVID-19 = Coronavirus disease-2019.

**Table 2:** Electrocardiogram changes of the two cases that presented to a tertiary care hospital in Muscat, Oman in 2019

Findings	Case 1	Case 2
P-wave	Peaked and wide, abnormal	Peaked and wide, abnormal
ST-T changes	No	Yes
Axis	Right axis deviation	Normal
RVH	No	No
LVH	No	No
QTc* in msec	440	450
Any other abnormalities	Bi-atrial enlargement	Bi-atrial enlargement

RVH = right ventricular hypertrophy; LVH = left ventricular hypertrophy; QTc = corrected

* QT interval for heart rate.

**Table 3:** Echocardiography findings of the two cases that presented to a tertiary care hospital in Muscat, Oman in 2019

Findings	Case 1	Case 2
RA size	Dilated	Dilated
LA size	Dilated	Dilated
LVEF in %	50	30–35
TR in mmHg	Severe – 15	Moderate - 50
MR	Not present	Moderate
Lateral wall E’ in cm/s	16	6
Lateral wall A’ in cm/s	3	5
Hepatic vein A wave reversal in m/s	0.5	Not available
MV inflow E wave velocity in m/s	0.35	0.77
MV inflow A wave velocity in m/s	0.27	0.35
MV E/A	1.31	2.2
Pulmonary vein A wave doppler	Present	Present
MV deceleration time in ms	107	190
Pericardial effusion	Not present	Not present
Impression	Mixed type of left ventricular non-compaction with severe	Mildly hypertrophied infiltrated left ventricle with restrictive cardiomyopathy moderately depressed function.

RA = right atrium; LA = left atrium; LVEF = left ventricle ejection fraction; TR = tricuspid regurgitation; MR = mitral regurgitation; MV = mitral valve.
